# Modulation of Cecal Microbiota and Inflammatory-Immune Pathways Underlies the Reduced Eliminations but Unchanged Growth in Broilers Fed a Compound Probiotic

**DOI:** 10.3390/ani16213385

**Published:** 2026-10-27

**Authors:** Junyi Li, Xinli Gong, Tian Zhou, Junjin Deng, Shuyang Liu, Jing Liu, Tingting Lu, Cheng Ran, Jiaquan Li, Wenqing Zuo, Zhilin Wang, Jiazhou Li

**Affiliations:** 1 Circular Agriculture Research Center, Heyuan Branch, Guangdong Laboratory for Lingnan Modern Agriculture, Heyuan 517000, China; leizeonngai1206@163.com (J.L.);; 2 Agro-Biological Gene Research Center, State Key Laboratory of Livestock and Poultry Breeding, Guangdong Academy of Agricultural Sciences, No. 20 Jinying Road, Tianhe District, Guangzhou 510640, China; 3 School of Low-Altitude Technology and Engineering, Guangdong Polytechnic Normal University, Heyuan 517500, China

**Keywords:** *Lactobacillus salivarius*, *Lactobacillus acidophilus*, *Lactobacillus reuteri*, yellow-feathered broiler, growth performance, serum biochemistry, intestinal morphology, gut microbiota

## Abstract

With the global restriction on antibiotic growth promoters in poultry production, probiotics have emerged as promising alternatives. This study evaluated the effects of a compound probiotic formulation consisting of *Lactobacillus salivarius*, *Lactobacillus acidophilus*, and *Lactobacillus reuteri* at a ratio of 38:9:1 on growth performance and gut microbiota in Lingnan yellow-feathered broilers. A total of 700 one-day-old broilers were randomly assigned to a control group (basal diet) or a probiotic group (basal diet plus 1.45 × 10^9^ CFU/L of the compound probiotic in drinking water, administered intermittently from 22 to 63 days of age). Growth performance, serum biochemical parameters, and intestinal morphology were measured, and 16S rRNA sequencing was performed on fecal, ileal, and cecal samples. Results showed that probiotic supplementation did not significantly affect growth performance, serum biochemical indices, or intestinal villus morphology. However, elimination rate during 43–63 days and over the entire experimental period was significantly reduced in the probiotic group. 16S rRNA sequencing revealed that the probiotic group exhibited significantly increased cecal microbial richness without alterations in community evenness, along with decreased *Lactobacillus* abundance in the ileum and increased *Akkermansia* abundance in the cecum. Functional prediction of cecal microbiota showed that 84 pathways, including inflammation- and immunity-related pathways such as the NOD-like receptor signaling pathway and multiple core functional categories, were significantly down-regulated, while only three pathways were significantly up-regulated. Collectively, although the compound probiotic failed to improve growth performance, it effectively reduced elimination rate, potentially through enrichment of beneficial *Akkermansia* and modulation of intestinal inflammation-related pathways.

## Simple Summary

As the global poultry industry phases out antibiotics used to boost bird growth, probiotics—live beneficial bacteria that support gut health—have emerged as a widely explored alternative. However, their effectiveness varies greatly depending on the bacterial strains used, strain combination ratio, dosage, initiation timing of supplementation, administration methods, chicken breeds, etc. This study evaluated a three-strain *Lactobacillus* probiotic compound given intermittently in drinking water to fast-growing yellow-feathered broilers. The results showed that the probiotic did not improve growth rate or intestinal tissue structure, but significantly reduced elimination rate in the later growing stage and across the entire rearing period. This health benefit may be linked to higher levels of beneficial gut bacteria and reduced mild intestinal inflammation. These findings offer practical guidance for probiotic use in antibiotic-free poultry production, supporting flock health and farming efficiency.

## 1. Introduction

Over the past several decades, the poultry industry has achieved substantial improvements in production efficiency through continuous advances in genetic selection, feeding management, and nutritional regulation [1]. During this process, antibiotics have been widely used as growth promoters in poultry feed to improve growth performance, reduce disease incidence, and enhance feed conversion efficiency [2]. However, prolonged and inappropriate use of antibiotics has led to the emergence of bacterial resistance and drug residues in animal products, posing a serious threat to public health [3]. In 2006, the European Union took the lead in banning the use of antibiotic growth promoters in animal production [4], followed by similar restrictive measures in many countries and regions [2]. Although the implementation of antibiotic bans has mitigated the risk of antimicrobial resistance transmission to some extent, it has also brought about negative consequences such as reduced growth performance and increased intestinal health problems [2]. Therefore, the search for safe, effective, and environmentally friendly alternatives to antibiotics has become one of the key research priorities in poultry nutrition [5].

Among the various antibiotic alternative strategies, probiotics have attracted widespread attention due to their low production cost and broad applicability [6]. According to the definition by the International Scientific Association for Probiotics and Prebiotics, probiotics are “live microorganisms that, when administered in adequate amounts, confer a health benefit on the host” [7]. Studies have demonstrated that probiotics exert their beneficial effects through multiple mechanisms, including regulating and stabilizing the gut microbial community, inhibiting pathogen colonization through competitive exclusion and production of antimicrobial metabolites, secreting digestive enzymes to enhance nutrient digestibility, and stimulating beneficial fermentation processes [8,9]. In addition, probiotics can adhere to the intestinal mucosa to form a physical barrier, preventing pathogen adhesion, while also maintaining intestinal health by modulating intestinal barrier function and the expression of immune-related genes [10]. Numerous studies have confirmed that probiotic supplementation can increase body weight gain and feed conversion efficiency in broilers, as well as enhance disease resistance and reduce disease susceptibility through immunomodulatory effects [11,12].

In terms of probiotic strain selection, *Lactobacillus* is one of the dominant bacterial groups in the poultry gut and is also the most widely applied probiotic category [13]. Among them, *Lactobacillus salivarius*, *Lactobacillus reuteri*, and *Lactobacillus acidophilus* are three *Lactobacillus* species that have been relatively well studied [14]. *L. salivarius* is an important member of the chicken gut microbiota and has been shown to effectively inhibit the colonization of *Salmonella enteritidis* in the intestine [15]. Studies have shown that *L. salivarius* D5 can significantly increase the average daily gain of broilers, increase spleen index and serum IgM levels, and promote the villus development of the jejunum and ileum as well as intestinal IL-10 expression [16]. Moreover, *L. salivarius* can reshape the cecal microbial community structure, increase the ratio of *Firmicutes* to *Bacteroidetes* and the relative abundance of beneficial genera [16]. Another study also confirmed that *L. salivarius* RBL50 can improve body weight gain and average daily gain of broilers, reduce the feed-to-gain ratio, increase jejunal villus height and villus height-to-crypt depth ratio, up-regulate the expression of intestinal tight junction proteins claudin-1 and ZO-1, and enhance cellular immune function [17].

*L. reuteri* also exhibits favorable probiotic properties. Studies have found that, similarly to *L. salivarius* RBL50, *L. reuteri* SW23 can significantly improve the growth performance and intestinal morphology of broilers, and can also reduce serum total cholesterol concentration [17]. In a necrotic enteritis challenge model, *L. reuteri* postbiotics improved body weight gain and average daily gain of challenged broilers, reduced plasma diamine oxidase and lysozyme levels, increased jejunal villus height and the expression of Claudin-1 and Mucin2 in the ileum, while down-regulating the expression of pro-inflammatory cytokines IL-1β and IFN-γ in the ileum [18]. Furthermore, dietary supplementation with *L. reuteri* SL001 significantly increased the total weight gain and average daily gain of broilers at 42 days of age, increased serum catalase (CAT) levels and total antioxidant capacity (T-AOC), improved jejunal and ileal villus height and villus height-to-crypt depth ratio, modulated the cecal microbiota composition, and enriched beneficial bacterial communities associated with growth and health [19]. In addition, *L. reuteri* can alleviate lipopolysaccharide (LPS)-induced intestinal mucosal damage in broilers by activating the Wnt/β-catenin signaling pathway to stimulate the expansion of intestinal stem cells [20].

*L. acidophilus* is also a commonly used probiotic strain in poultry production. Studies have shown that supplementation with *L. acidophilus* significantly reduced average daily feed intake, feed-to-gain ratio, and abdominal fat percentage, while increasing serum high-density lipoprotein cholesterol and IL-2 and IL-4 contents, and decreasing lipopolysaccharide (LPS) levels, suggesting that it improves feed efficiency and immune function mainly through regulating nutrient metabolism and inflammatory responses [21].

Although the respective probiotic effects of these three *Lactobacillus* species have been extensively reported, studies on the probiotic effects of their combination at specific ratios on broilers, especially regarding the microbial community structure and function at different growth stages and different intestinal sites, remain relatively limited. Moreover, the relationship between the mode of probiotic administration (such as concentration, frequency, and cycle of drinking water supplementation) and its regulatory effects on the host microbial community is complex, and the results across different studies are inconsistent [22,23].

Based on the above background, the present study used Lingnan yellow-feathered broilers as experimental animals and formulated a composite probiotic preparation with *L. salivarius*, *L. acidophilus*, and *L. reuteri* at a live bacterial count ratio of 38:9:1. The preparation was intermittently administered via drinking water from 22 days of age until slaughter at 63 days of age. The aim was to systematically evaluate the effects of this composite probiotic preparation on growth performance, gut microbial community diversity and structure, intestinal morphology, and intestinal KEGG functional pathways, so as to provide a scientific basis for the rational application of composite *Lactobacillus* preparations in broiler production.

## 2. Materials and Methods

### 2.1. Probiotic Strains

The composite probiotic formulation used in this experiment was prepared by combining *Lactobacillus salivarius*, *L. acidophilus*, and *L. reuteri* at a live bacterial count ratio of 38:9:1. This ratio was designed based on our previously unpublished data on the intestinal microbiome of healthy Lingnan yellow-feathered broilers exhibiting superior growth performance, in which the relative abundances of the corresponding *Lactobacillus* species in the ileal microbiota naturally approximated this proportion. Among these, *L. reuteri* LR1 was isolated and preserved by our laboratory, originally obtained from the ileal contents of healthy pigs raised in a large-scale commercial farm in Guangzhou, China. *L. acidophilus* was purchased from Hubei Xinkang Pharmaceutical Chemical Co., Ltd. (Wuhan, China), and *L. salivarius* was purchased from Zhengzhou Hehe Bioengineering Technology Co., Ltd. (Zhengzhou, China).

### 2.2. Experimental Animals, Diets, and Experimental Design

A total of 700 one-day-old healthy Lingnan yellow-feathered broilers (fast-growing type) with equal numbers of males and females were used in this experiment. There were no significant differences in initial body weight between groups (*p* > 0.05). The experiment was conducted at a commercial farm in Baiyun District, Guangzhou, Guangdong Province. The birds were vaccinated according to standard immunization procedures and then transferred to experimental animal facilities for routine rearing. After a 3-day adaptation period, during which the health status of all birds was confirmed, the broilers were randomly assigned into two treatment groups at 4 days of age based on similar body weight, with 5 replicates per group and 70 birds per replicate, totaling 10 pens. The formal experimental period lasted 63 days (9 weeks). The treatment groups were designed as follows: (1) Control group (Ctrl): birds received a basal diet and regular drinking water throughout the entire period, without any antibiotic or probiotic supplementation; (2) Probiotic group (Test): birds received the basal diet and regular drinking water from 1 to 21 days of age, without any antibiotic or probiotic supplementation; from 22 days of age onward, the composite probiotic was added to the drinking water at a final concentration of 1.45 × 10^9^ CFU/L. Based on this target concentration and the live bacterial count ratio of 38:9:1 among *L. salivarius*, *L. acidophilus*, and *L. reuteri*, the amount of each strain to be added per liter of medicated water was calculated accordingly. The probiotic was administered continuously for 4 days followed by a 3-day withdrawal period, and this cycle was repeated until the end of the experiment.

The basal diet (Table 1) was formulated to meet the nutritional requirements of broilers at each growth stage, following the Feeding Standard of Chicken (NY/T 33-2004), Nutrient Requirements for Yellow Chickens (NY/T 3645-2020), and Nutrient Requirements of Poultry: Ninth Revised Edition, 1994 (NRC, 1994). During the experimental period, the birds had free access to feed and water and were reared in cages with a lighting schedule of 20 h light and 4 h darkness maintained throughout the entire period. The house temperature was controlled using heating equipment.

Bird health status was monitored once daily by trained caretakers who were blinded to treatment assignment. Predefined humane removal endpoints were established prior to the trial in accordance with the institutional animal welfare guidelines: birds were eliminated (removed from the experimental pen and transferred to separate isolated rearing pens for separate care) when they exhibited any of the following persistent signs for ≥24 h: (i) severe depression, recumbency, or complete cessation of feed and water intake; (ii) inability to stand or ambulate; (iii) severe respiratory distress or neurological signs; or (iv) body weight < 70% of the age-matched pen mean accompanied by progressive emaciation. Birds that died spontaneously within experimental pens without meeting removal criteria during monitoring were recorded separately as natural mortality. All elimination decisions were documented daily, including the date, pen, and primary clinical reason for removal, and all records were cross-checked by a second veterinarian to ensure decision consistency. Body weight was measured after fasting (with free access to water) at 20:00 on the day before 21, 42, and 63 days of age, and weighing was conducted at 08:00 the next morning on a replicate basis.

### 2.3. Sample Collection and Processing

At 31 days of age (mid-trial) and 63 days of age (end of trial), one fresh fecal sample was collected from each replicate pen, yielding 5 fecal samples per group at each time point (10 samples per group across the two time points; 20 fecal samples in total, designated F31 and F63, respectively). At 63 days of age, two birds per pen were randomly selected near the average body weight of the pen and euthanized by cervical dislocation. Immediately after slaughter, the jejunum, ileum, and cecum were aseptically dissected. For microbiome analysis, the ileal and cecal contents were collected from each bird, yielding 10 ileal samples and 10 cecal samples per group (40 intestinal content samples in total, designated I63 and C63, respectively). Together with the 20 fecal samples, a total of 60 samples were subjected to 16S rRNA gene sequencing. All samples were immediately placed into sterile cryogenic vials, snap-frozen in liquid nitrogen, and stored at −80 °C until further analysis (Novogene Co., Ltd., Beijing, China). For intestinal morphology analysis, the sampling and measurement procedures were performed according to the method of Chai et al. [19] with minor modifications. A 2 cm segment from the mid-jejunum and a 2 cm segment from the mid-ileum were collected from each bird (20 birds per group, 40 intestinal segments in total). The tissue segments were gently flushed with ice-cold phosphate-buffered saline (PBS, pH 7.4; Servicebio, Wuhan, China) to remove digesta, and fixed in 4% paraformaldehyde (Servicebio, Wuhan, China) for 48 h at room temperature. After fixation, the samples were dehydrated through a graded ethanol series, embedded in paraffin, sectioned, deparaffinized with xylene and a descending ethanol series, and subsequently stained with hematoxylin and eosin (HE; Servicebio, Wuhan, China). Villus height and crypt depth were measured using a light microscope equipped with Saiviewer 3.01 software (Servicebio, Wuhan, China). Villus height was defined as the distance from the tip to the base of the villus, and crypt depth was measured from the base of the villus to the base of the crypt; the villus-to-crypt (V/C) ratio was calculated accordingly. For serum biochemical analysis, blood samples were collected via wing vein puncture into additive-free serum tubes (Guangzhou Improve Medical Instruments Co., Ltd., Guangzhou, China) (3 mL per bird; 20 samples in total) and immediately placed on ice. After standing at room temperature, the samples were centrifuged to separate serum. Serum urea nitrogen (SUN), creatinine (CREA), triglycerides (TG), and total cholesterol (TC) were measured using an automatic biochemical analyzer (Rayto Life Analytical Instruments Co., Ltd., Shenzhen, China) with commercial kits (Rayto/Servicebio) following the manufacturer’s instructions. All samples were stored properly and protected from repeated freeze–thaw cycles. Euthanasia and all experimental procedures were conducted in accordance with the guidelines of the Institutional Animal Care and Use Committee.

### 2.4. Determination of Growth Performance Parameters

Based on fasting body weight and feed intake and daily health monitoring records (including spontaneous mortality and elimination events) at each time point, the average daily gain (ADG), total weight gain (TWG), average daily feed intake (ADFI), total feed consumption (TFC), feed conversion rate (FCR), survival rate (SR), and elimination rate (ER) were calculated for different growth stages and the entire experimental period. All calculations were performed on a per-pen basis as defined below:

Average daily gain (ADG, g/d) = (average body weight of broilers at the end of the period − average body weight of broilers at the beginning of the period)/number of days in the period;

Total weight gain (TWG, kg) = (total body weight of broilers at the end of the period + total body weight of spontaneously dead broilers during the period + total body weight of eliminated broilers during the period) − total body weight of broilers at the beginning of the period;

Average daily feed intake (ADFI, g/d per bird) = (total feed consumption during the period − feed consumption of spontaneously dead and eliminated broilers during the period)/(number of broilers at the end of the period × number of days in the period);

Total feed consumption (TFC, kg) = residual feed weight at the end of the previous period + total feed provided during the period − residual feed weight at the end of the current period;

Feed conversion ratio (FCR) = TFC/TWG;

Survival rate (SR, %) = [(number of live broilers at the beginning of the period − number of spontaneously dead broilers during the period)/number of live broilers at the beginning of the period] × 100%;

Elimination rate (ER, %) = (number of birds eliminated during the period/number of live birds at the beginning of the period) × 100%

### 2.5. 16S rRNA High-Throughput Sequencing and Analysis of Gut Microbiome

#### 2.5.1. Microbial Genomic DNA Extraction, Library Construction, and High-Throughput Sequencing

Total microbial genomic DNA was extracted from ileal and cecal content samples using the CTAB-SDS method. DNA integrity was assessed by 2% agarose gel (TSingKe, Beijing, China) electrophoresis, and DNA concentration and purity were quantified using an SMA4000 microspectrophotometer (Beijing Starwood Technology Co., Ltd., Beijing, China). Qualified genomic DNA was diluted to 1 ng/μL as a template for PCR amplification. The bacterial 16S rRNA gene V4 region was amplified using primers 515F (5′-GTGCCAGCMGCCGCGGTAA-3′) and 806R (5′-GGACTACHVGGGTWTCTAAT-3′). The eukaryotic 18S rRNA gene was amplified using primers 528F (5′-GCGGTAATTCCAGCTCCAA-3′) and 706R (5′-AATCCRAGAATTTCACCTCT-3′). The fungal ITS region was amplified using primers ITS5-1737F (5′-GGAAGTAAAAGTCGTAACAAGG-3′) and ITS2-2043R (5′-GCTGCGTTCTTCATCGATGC-3′). All primers were barcoded with specific tags. The PCR products were purified, and sequencing libraries were constructed using the TruSeq^®^ DNA PCR-Free Library Preparation Kit (Illumina Inc., San Diego, CA, USA). After quality control, the libraries were sequenced on the Illumina NovaSeq 6000 platform at Beijing Novogene Technology Co., Ltd.

#### 2.5.2. Sequencing Data Quality Control and Taxonomic Annotation

The raw reads obtained from sequencing were subjected to systematic quality control to remove adapter sequences, low-quality sequences, short invalid fragments, and sequences of host and feed origin. The high-quality clean tags obtained were used for subsequent bioinformatics analysis. Operational taxonomic units (OTUs) were clustered from the clean tags of all samples using Uparse v7.0.1001 software at 97% sequence identity, and the most abundant sequence in each OTU was selected as the representative sequence. Taxonomic annotation was performed by aligning the OTU representative sequences against the SILVA 138.1 SSU rRNA database using the Mothur method, with a confidence threshold of 0.8–1.0, yielding taxonomic information at seven levels: kingdom, phylum, class, order, family, genus, and species. The community composition at each taxonomic level was determined for each sample. Multiple sequence alignment was performed using MUSCLE v3.8.31 to construct a phylogenetic tree of all OTU representative sequences. Functional annotation and data normalization of the microbial communities were performed by integrating the KEGG and eggNOG databases based on the taxonomic annotation results.

#### 2.5.3. Analysis of Microbial Community Diversity and Differences

Microbiome data were analyzed and visualized using R 2.15.3 software. Alpha diversity indices of microbial communities, including the number of observed OTUs, Chao1 index, ACE index, Simpson index, and Shannon index, were calculated using QIIME 2. Intergroup comparisons were performed using Tukey’s test and the Wilcoxon test to comprehensively evaluate species richness and evenness. Principal coordinates analysis (PCoA) based on the Bray–Curtis distance matrix was performed to assess the overall differences and clustering characteristics of microbial community structure between treatment groups. Permutational multivariate analysis of variance (PERMANOVA) was performed to test the statistical significance of differences in overall microbial community composition between dietary treatments. Linear discriminant analysis effect size (LEfSe) was used to evaluate the contribution of differential taxa to intergroup differences.

### 2.6. Statistical Analysis

Phenotypic data, including growth performance and biochemical parameters, were analyzed by one-way analysis of variance (ANOVA) using GraphPad Prism 8.0.2 software. Intergroup comparisons of means were performed using unpaired t-tests. The results are expressed as means ± standard deviation (SD), and differences at *p* < 0.05 were considered statistically significant. The pen was defined as the experimental unit for all phenotypic data.

## 3. Results

### 3.1. Growth Performance

Water consumption and probiotic intake in the Test group are recorded and estimated. No probiotic was administered during 1–21 d. The total water intake containing probiotics was 852.86 L during 22–42 d and 1198.71 L during 43–63 d. The estimated daily probiotic intake per bird was 0.29 × 10^9^ CFU during 22–42 d, 0.42 × 10^9^ CFU during 43–63 d, and 0.36 × 10^9^ CFU over the entire experimental period (Supplementary Table S1).

**Figure 1:**
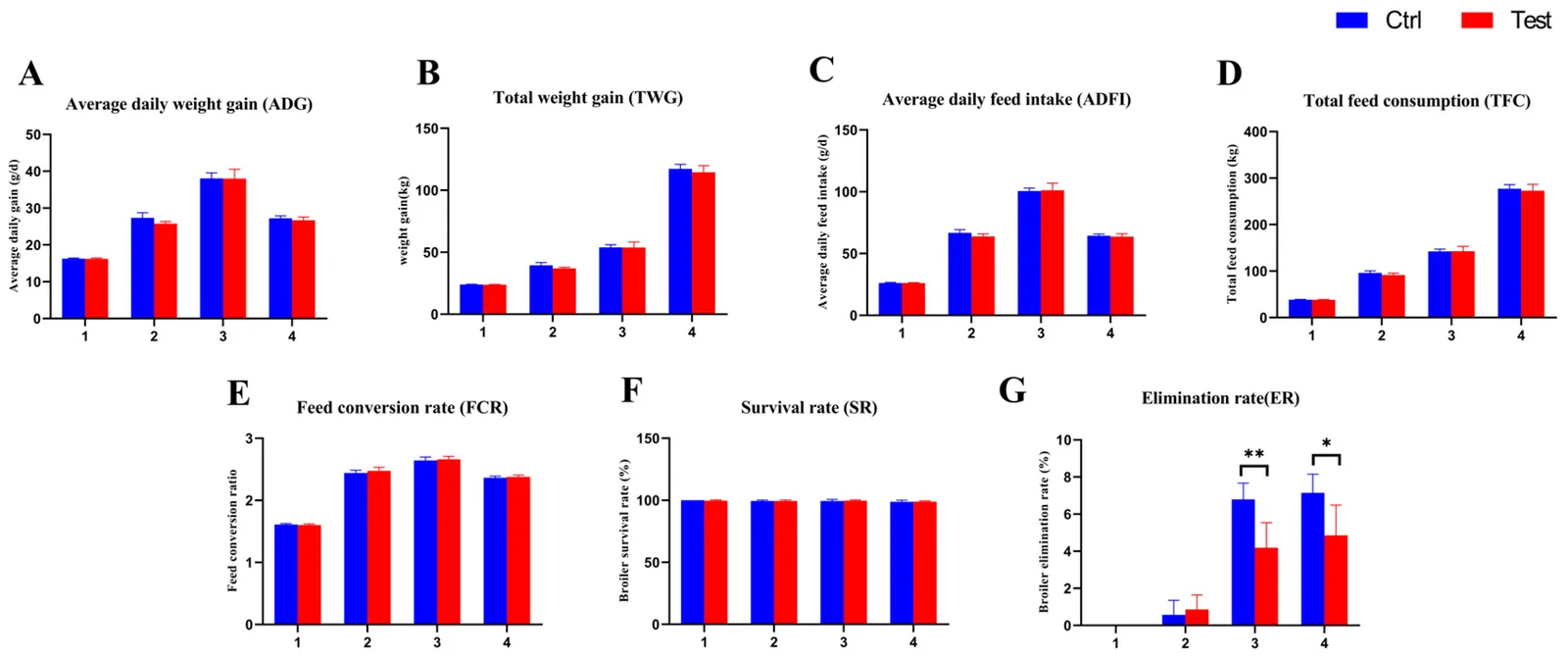
Growth performance parameters of yellow-feathered broilers fed compound probiotics at different growth stages. (**A**) Average daily gain (ADG); (**B**) Total weight gain (TWG); (**C**) Average daily feed intake (ADFI); (**D**) Total feed consumption (TFC); (**E**) Feed conversion rate (FCR); (**F**) Survival rate (SR); (**G**) Elimination rate (ER). Asterisks indicate significant differences between groups (* *p* < 0.05; ** *p* < 0.01).

Analysis of growth performance parameters of broilers in the Ctrl and Test groups at different growth stages and over the entire experimental period (Supplementary Table S2) revealed no significant differences in average daily gain (ADG) between the two groups at 1–21 d, 22–42 d, 43–63 d, or over the whole period (*p* > 0.05) (Figure 1). Although the ADG of the Test group was numerically lower than that of the Ctrl group during 22–42 d (25.78 vs. 27.34 g/d), the difference did not reach statistical significance (*p* = 0.0548) (Figure 1A). Similarly, total weight gain (TWG) showed no significant differences at any stage or over the whole period (*p* > 0.05), with a tendency for lower values in the Test group during 22–42 d (36.96 vs. 39.46 kg, *p* = 0.0525) (Figure 1B). No significant differences were observed in average daily feed intake (ADFI) (Figure 1C) or total feed consumption (TFC) (Figure 1D) at any stage or over the whole period (*p* > 0.05), although ADFI during 22–42 d tended to be slightly lower in the Test group (63.82 vs. 66.68 g/d, *p* = 0.0937). The feed conversion rate (FCR) also showed no significant differences between the two groups at any stage or over the whole period (*p* > 0.05) (Figure 1E). In terms of survival rate (SR), no significant differences were found between groups at any stage or over the whole period (*p* > 0.05) (Figure 1F). However, the elimination rate (ER) during 43–63 d was significantly lower in the Test group than in the Ctrl group (4.190 ± 1.347% vs 6.793 ± 0.8786%, *p* = 0.0068), and this significant difference was also observed over the entire experimental period (4.857 ± 1.629% vs 7.143 ± 1.010%, *p* = 0.0285) (Figure 1G). Specifically, no birds were eliminated from either group during the 1–21 d period. During both the 22–42 d and 43–63 d periods, all eliminated birds in both groups were eliminated due to persistent reduced feed intake, progressive weight loss, and lethargy.

**Figure 2:**
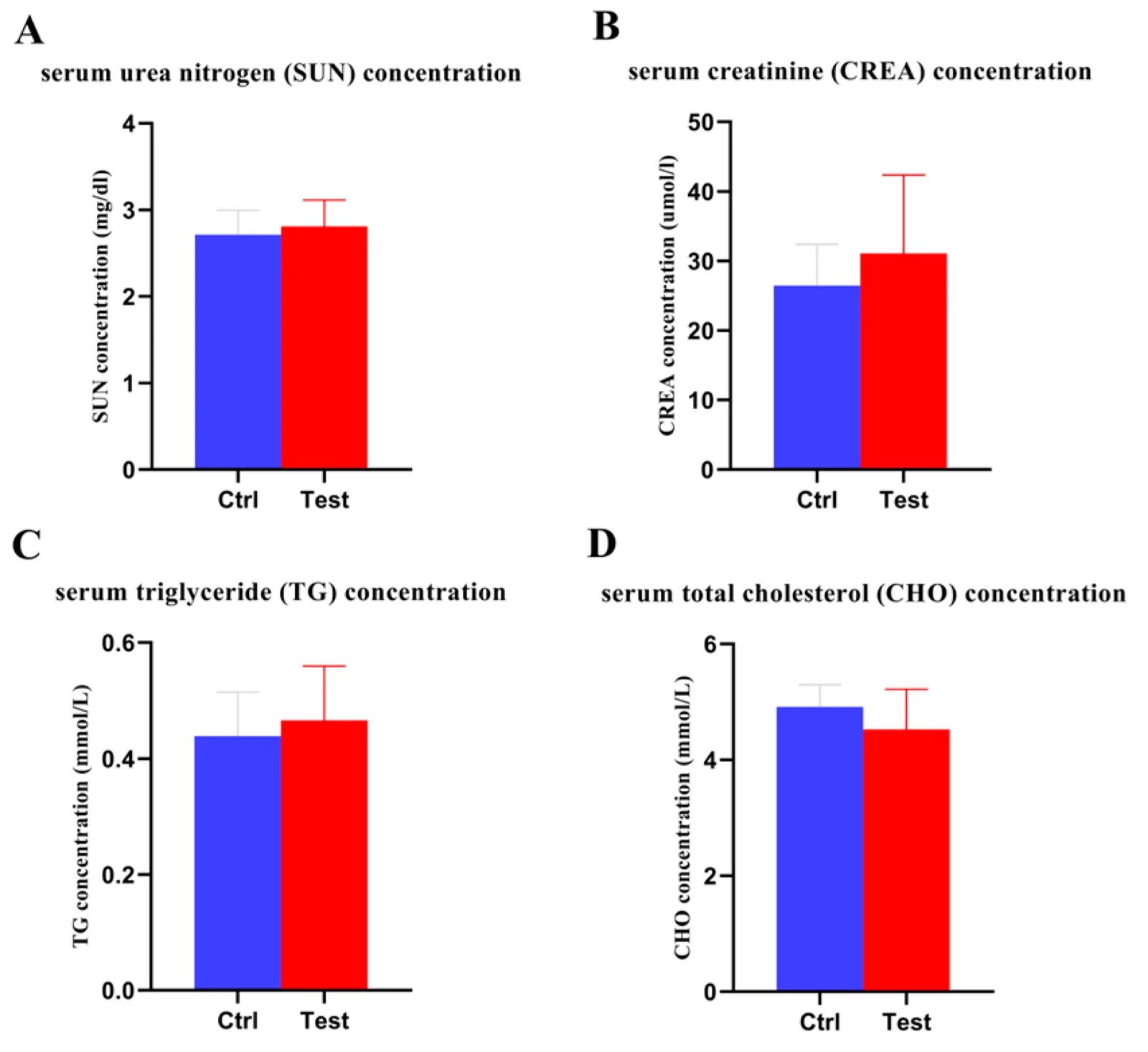
Serum biochemical parameters of yellow-feathered broilers fed compound probiotics on day 63. (**A**) serum urea nitrogen concentration; (**B**) serum creatinine concentration; (**C**) serum triglyceride concentration; (**D**) serum total cholesterol concentration.

In addition, serum biochemical parameters and key intestinal development indices were measured in the broilers from different treatment groups. For serum biochemical parameters, there were no significant differences (*p* > 0.05) in the contents of serum urea nitrogen (SUN), serum creatinine (CREA), serum triglyceride (TG), and serum total cholesterol (CHO) between the Ctrl group and the Test group (Figure 2). In terms of key intestinal development indicators, the villus-to-crypt ratios of the jejunum and ileum in the Test group were slightly higher than those in the Ctrl group, without significant differences (*p* > 0.05), whereas no significant differences (*p* > 0.05) were observed in villus height and crypt depth between the two groups (Table 2).

### 3.2. Richness and Diversity of Intestinal Microbes

**Figure 3:**
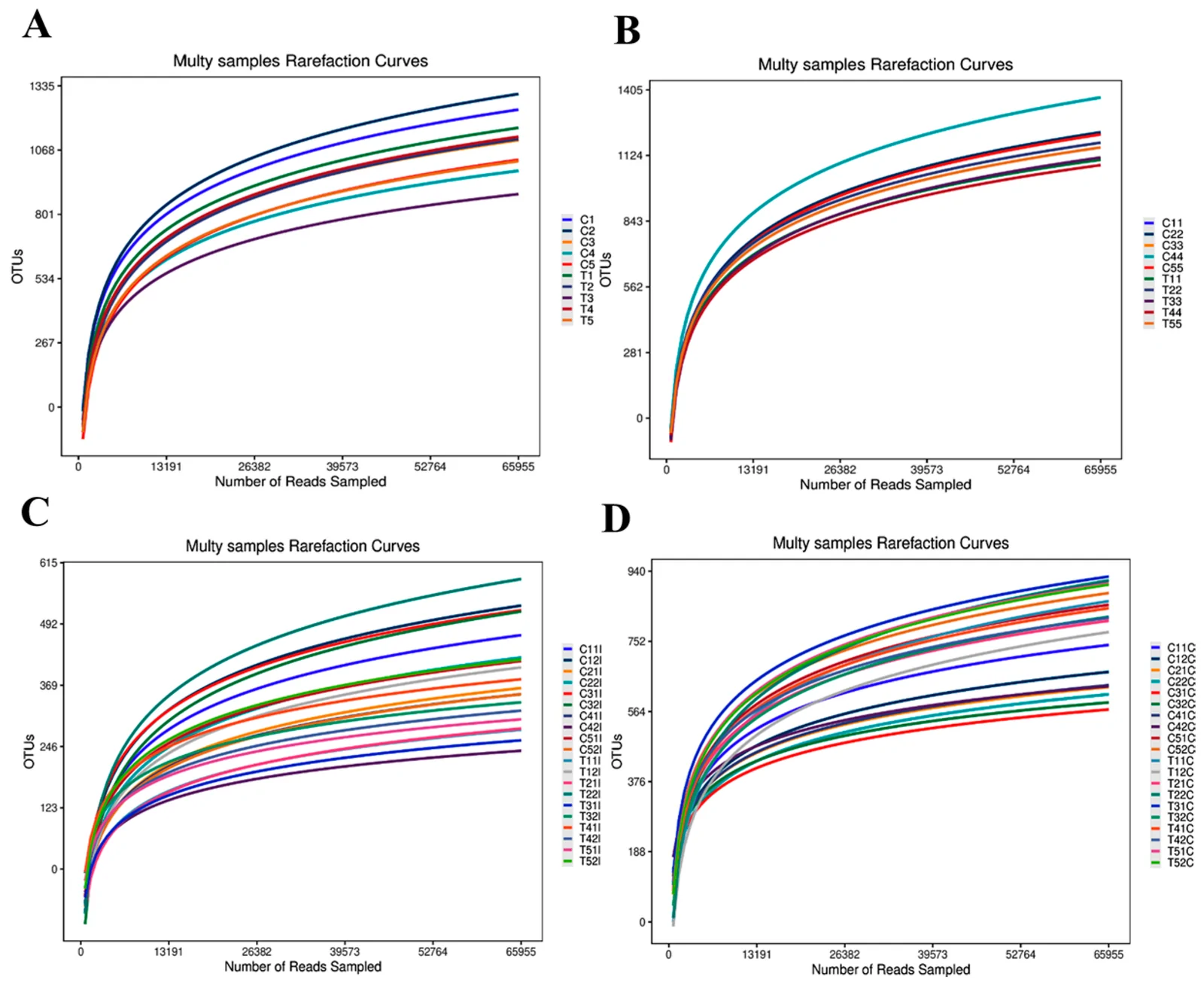
Rarefaction curves of microbial communities in feces and intestinal contents of yellow-feathered broilers fed compound probiotics at different sampling stages. (**A**) Fecal samples collected at the mid-term trial stage; (**B**) Fecal samples collected on day 63; (**C**) Ileal contents sampled on day 63; (**D**) Cecal contents sampled on day 63. C: blank control group (Ctrl); T: compound probiotic treatment group (Test).

**Figure 4:**
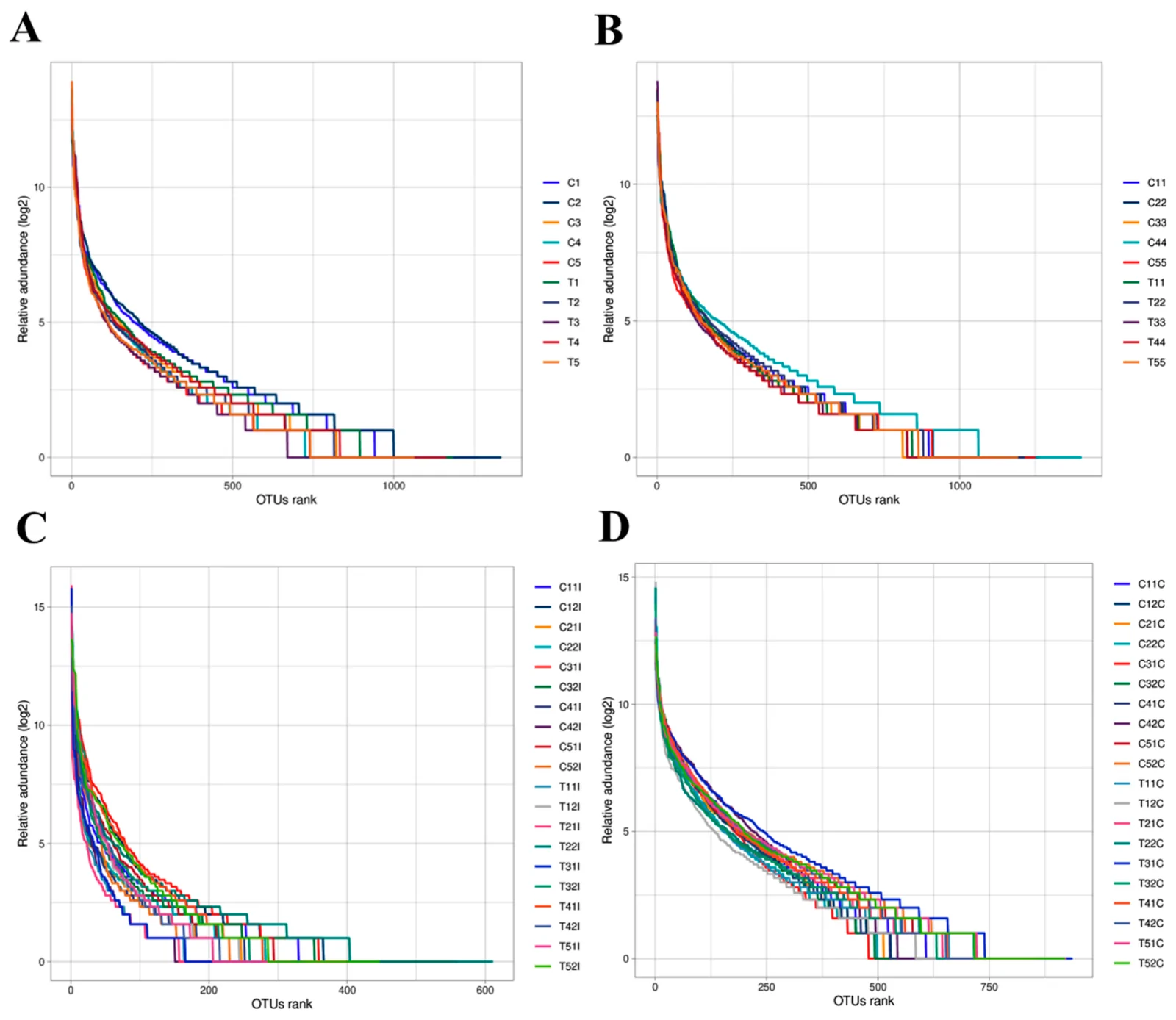
Rank-abundance curves of microbial communities in feces and intestinal contents of yellow-feathered broilers fed compound probiotics at different sampling stages. (**A**) Fecal samples collected at the mid-term trial stage (31 d); (**B**) Fecal samples collected on day 63; (**C**) Ileal contents sampled on day 63; (**D**) Cecal contents sampled on day 63. C: blank control group (Ctrl); T: compound probiotic treatment group (Test).

In this study, a total of 9,845,279 clean tags were obtained from 60 samples, with an average of 164,088 sequences per sample for subsequent analysis. As sequencing depth increased, species richness in each group increased markedly. Both the rarefaction curves (Figure 3) and rank-abundance curves (Figure 4) approached saturation, indicating that the sequencing depth in this study was sufficient to cover the vast majority of microbial taxa in the intestinal tract of broilers.

**Figure 5:**
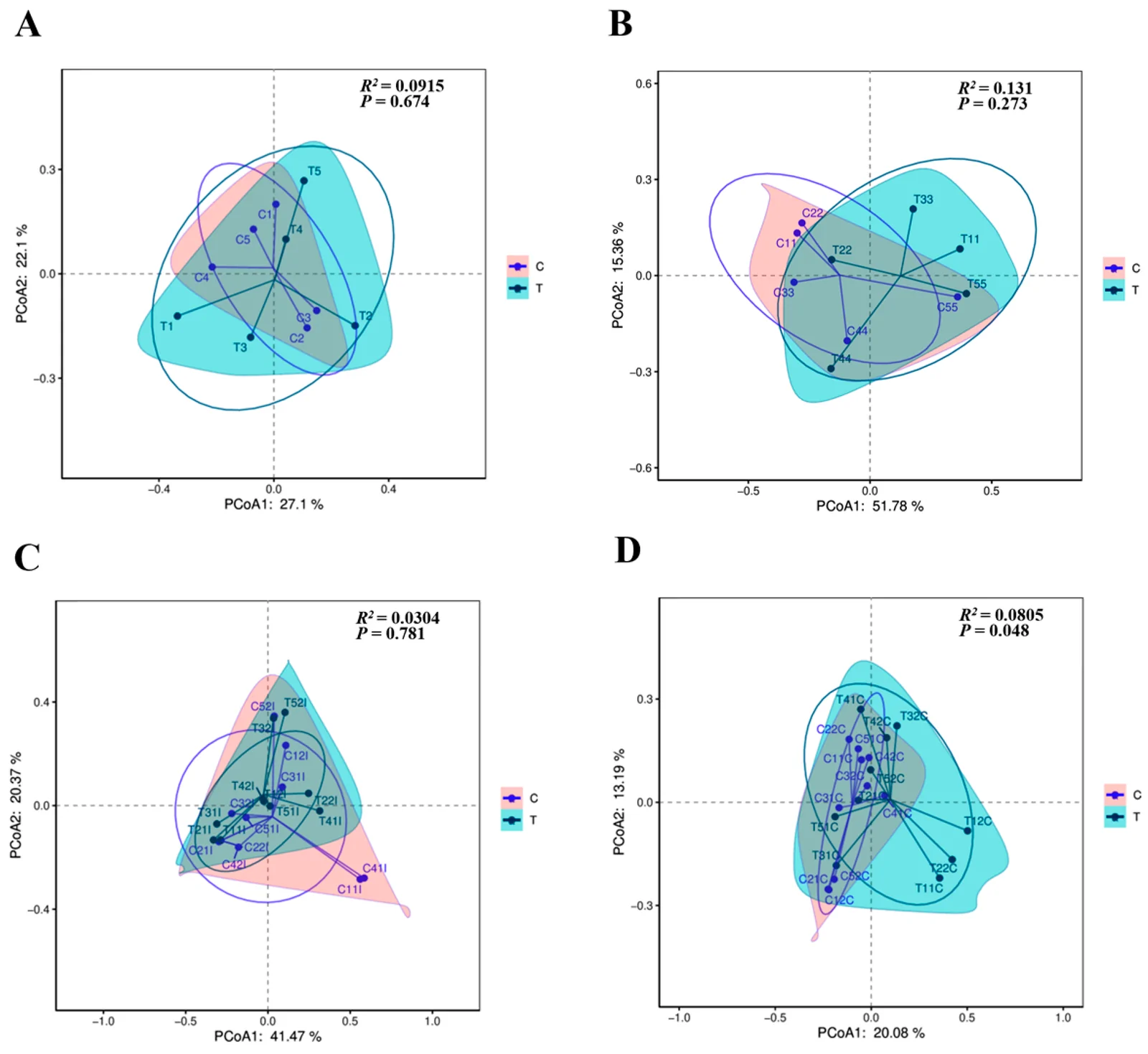
Principal coordinates analysis (PCoA) of microbial communities in feces and intestinal contents of yellow-feathered broilers fed compound probiotics at different sampling stages. (**A**) Fecal samples collected at the mid-term trial stage; (**B**) Fecal samples collected on day 63; (**C**) Ileal contents sampled on day 63; (**D**) Cecal contents sampled on day 63. The pink envelopes represent the blank control group, and the cyan envelopes represent the compound probiotic treatment group.

Principal coordinate analysis (PCoA) revealed that the response of the gut microbiota to probiotic intervention exhibited a temporal gradient and intestinal segment-specific differences. Compared with the highly intermixed community distribution observed in fecal samples at the mid-term stage (Figure 5A; *R*^2^ = 0.0915, *p* = 0.674), a tendency towards spatial differentiation between the two groups was observed in fecal samples on day 63 (Figure 5B; *R*^2^ = 0.131, *p* = 0.273), although this difference did not reach statistical significance. In contrast, the ileal microbiota remained indistinguishable between groups throughout the experimental period (Figure 5C; *R*^2^ = 0.0304, *p* = 0.781). Notably, a significant separation in community composition between the control and probiotic groups was detected in cecal contents at the end of the trial (Figure 5D; *R*^2^ = 0.0805, *p* = 0.048), indicating that the cecal microbiota was the most responsive intestinal segment to probiotic intervention.

**Figure 6:**
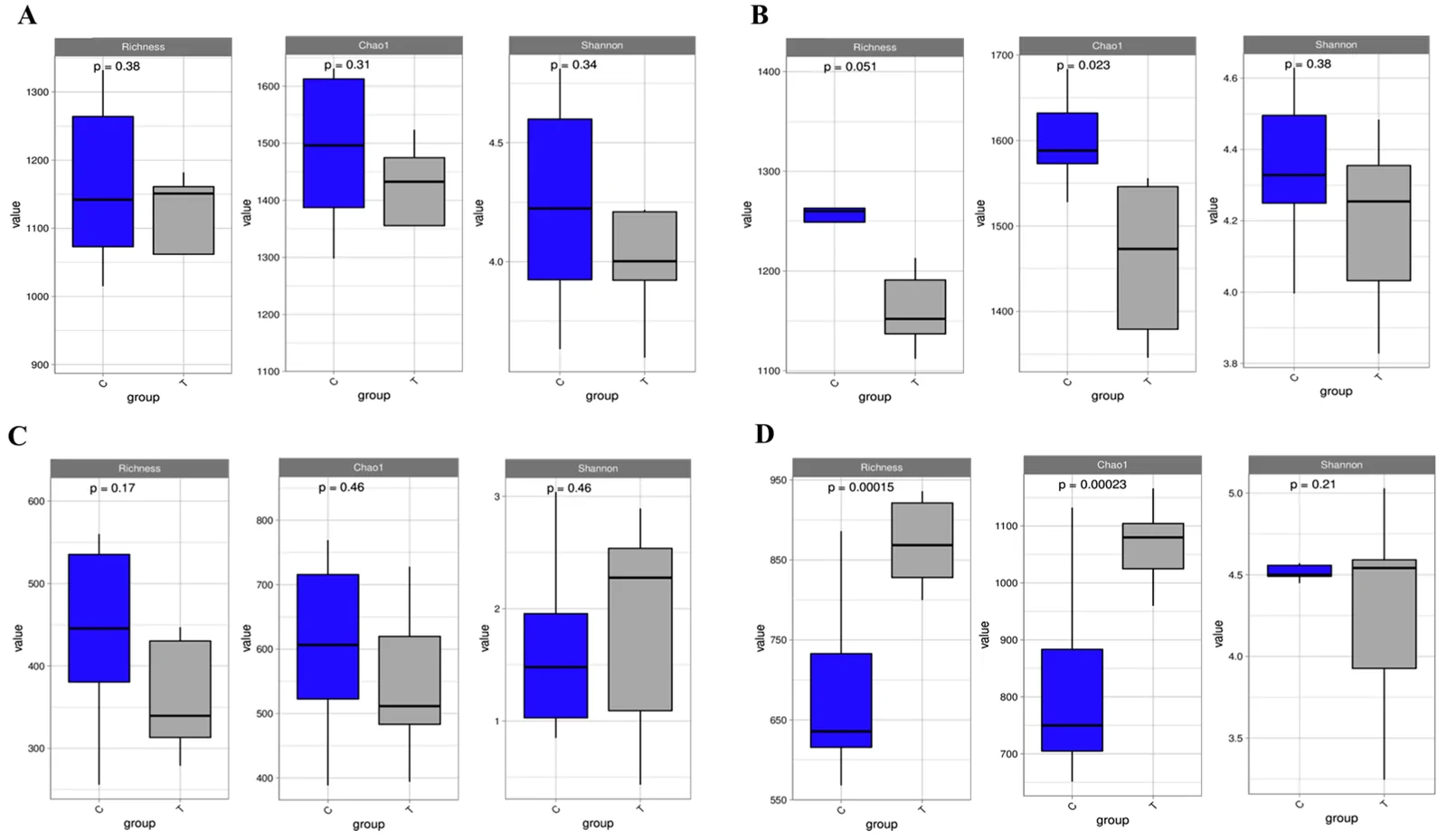
Alpha diversity indices of microbial communities in feces and intestinal contents of yellow-feathered broilers fed compound probiotics at different sampling stages. (**A**) Fecal samples collected at the mid-term trial stage (31 d); (**B**) Fecal samples collected on day 63; (**C**) Ileal contents sampled on day 63; (**D**) Cecal contents sampled on day 63. The blue boxes represent the blank control group (Ctrl), and the grey boxes represent the compound probiotic treatment group (Test). Within each panel, the left, middle, and right subpanels represent the Richness, Chao1, and Shannon indices, respectively. C: blank control group (Ctrl); T: compound probiotic treatment group (Test).

Alpha diversity analysis (Figure 6) revealed that the three indices (Richness, Chao1, and Shannon) exhibited varying patterns across different sample types. In cecal contents (Figure 6D), the Richness and Chao1 indices of the probiotic group were significantly higher than those of the control group (*p* < 0.05), whereas no significant difference was observed in the Shannon index between the two groups (*p* > 0.05). In contrast, no statistically significant differences were detected between the two groups for any of the Alpha diversity indices in mid-term fecal samples (Figure 6A), day-63 fecal samples (Figure 6B), or ileal contents (Figure 6C) (*p* > 0.05).

### 3.3. Composition of Intestinal Microbes

**Figure 7:**
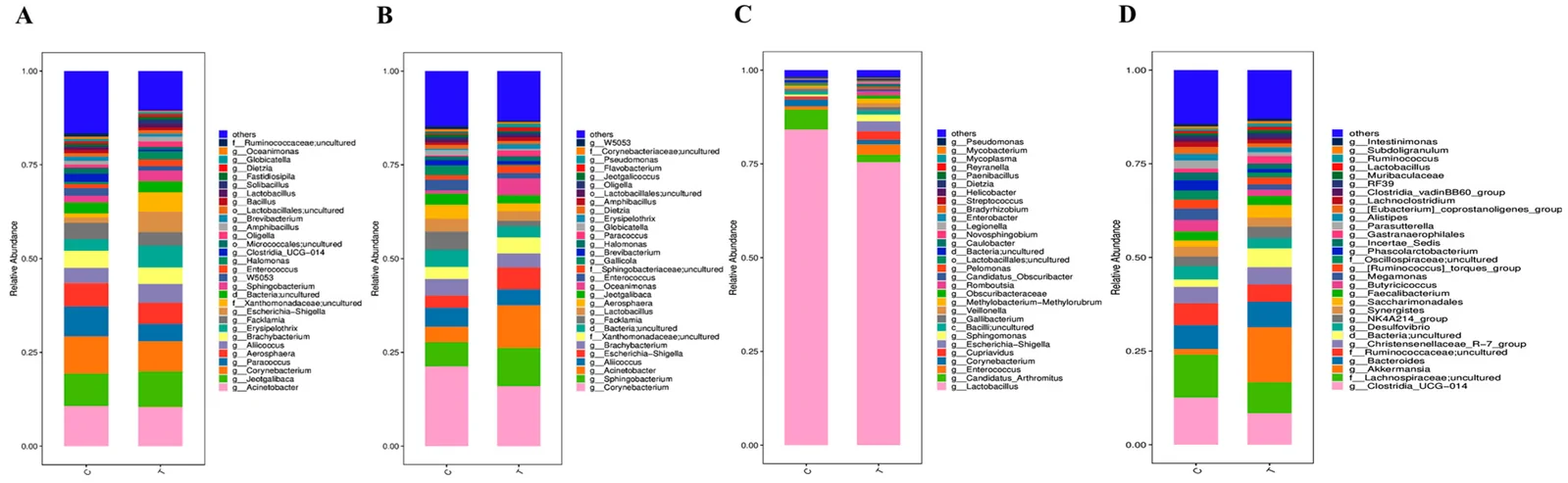
Relative abundance of microbial communities at the genus level in feces and intestinal contents of yellow-feathered broilers fed compound probiotics at different sampling stages. (**A**) Fecal samples collected at the mid-term trial stage; (**B**) Fecal samples collected on day 63; (**C**) Ileal contents sampled on day 63; (**D**) Cecal contents sampled on day 63. C: blank control group (Ctrl); T: compound probiotic treatment group (Test).

Figure 7 illustrates the relative abundance of microbial communities at the genus level across different groups. In fecal samples, the three most abundant genera were *Acinetobacter*, *Jeotgalibaca*, and *Corynebacterium* (Figure 7A,B). In ileal contents, the three most abundant genera were *Lactobacillus*, *Candidatus Arthromitus*, and *Enterococcus* (Figure 7C). In cecal contents, the three most abundant genera were *Clostridia_UCG-014*, *uncultured Lachnospiraceae*, and *Akkermansia* (Figure 7D).

**Figure 8:**
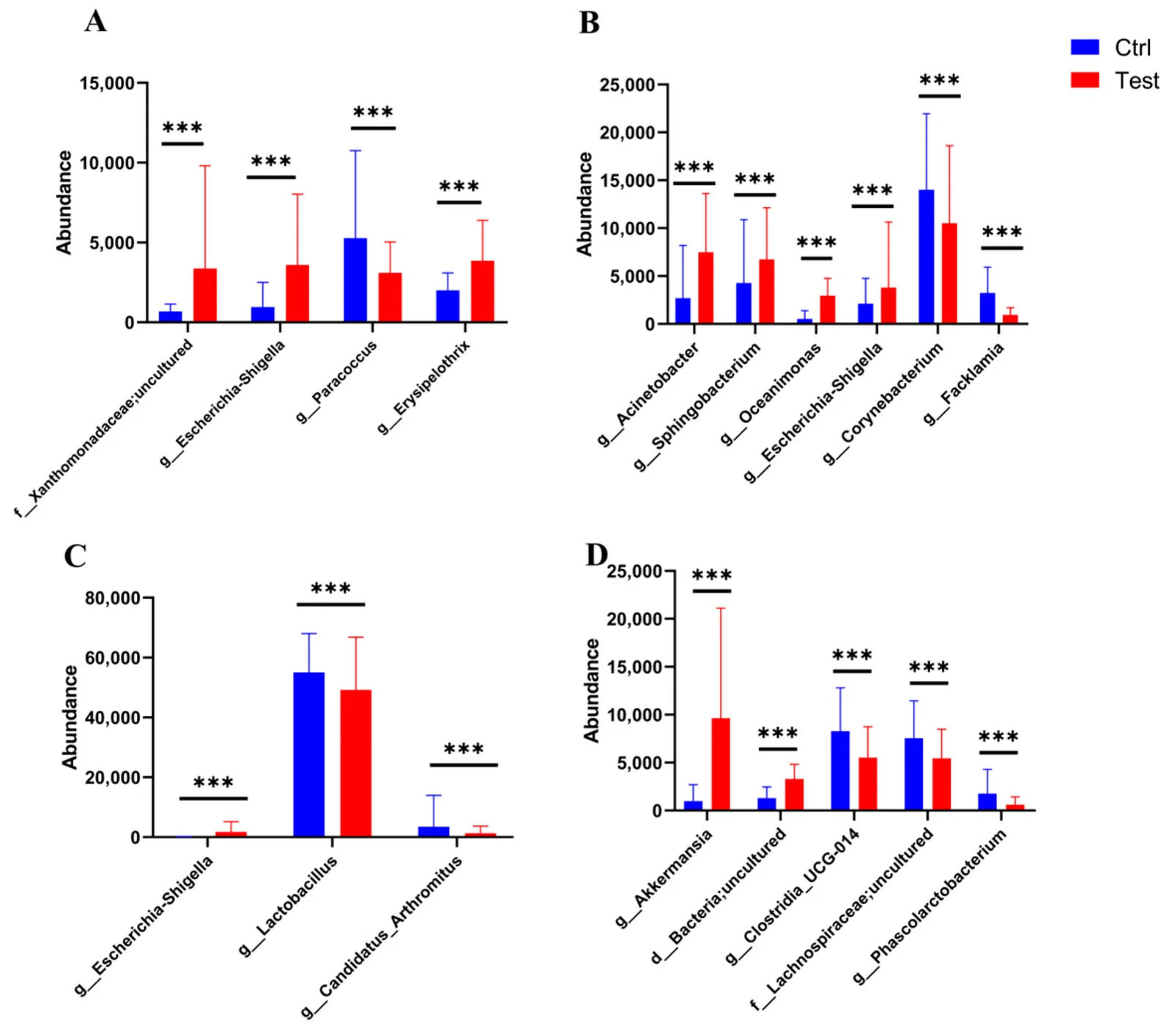
Differences in genus between groups in feces and intestinal contents of yellow-feathered broilers fed compound probiotics at different sampling stages. (**A**) Fecal samples collected at the mid-term trial stage (31 d); (**B**) Fecal samples collected on day 63; (**C**) Ileal contents sampled on day 63; (**D**) Cecal contents sampled on day 63. The blue bars represent genera enriched in the blank control group (Ctrl), and the red bars represent genera enriched in the compound probiotic treatment group (Test). Only genera with significant differences between groups (*p* < 0.05) are shown. *** *p* < 0.001 compared with the Ctrl group.

Student’s t-test was performed to identify genera with significantly different relative abundances between the control and probiotic groups. In mid-term (31 d) fecal samples, the probiotic group exhibited significantly increased abundances of *uncultured Xanthomonadaceae*, *Escherichia-Shigella*, and *Erysipelothrix*, along with a decreased abundance of *Paracoccus* (*p* < 0.001) (Figure 8A). In day-63 fecal samples, the probiotic group showed significant increases in *Acinetobacter*, *Sphingobacterium*, *Oceanimonas*, and *Escherichia-Shigella*, while abundances of *Corynebacterium* and *Facklamia* were significantly reduced (*p* < 0.001) (Figure 8B). In ileal contents at 63 d, *Escherichia-Shigella* was significantly increased in the probiotic group, whereas *Lactobacillus* and *Candidatus Arthromitus* were significantly decreased (*p* < 0.001) (Figure 8C). In cecal contents at 63 d, the probiotic group exhibited significant increases in *Akkermansia* and *uncultured Bacteria*, along with significant decreases in *Clostridia_UCG-014*, *uncultured Lachnospiraceae*, and *Phascolarctobacterium* (*p* < 0.001) (Figure 8D).

### 3.4. Predicted Function of Intestinal Microbes

**Figure 9:**
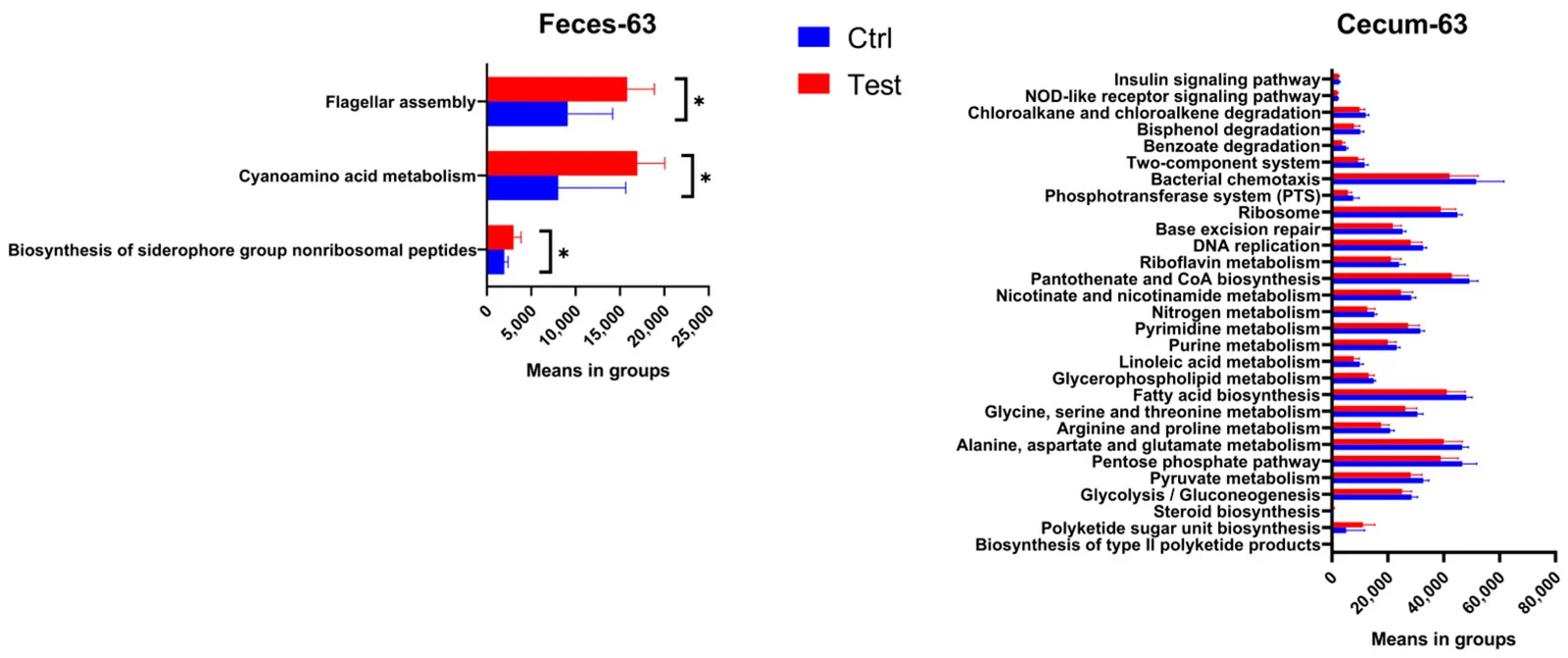
Differential KEGG pathways between groups. Feces-63: fecal samples collected on day 63; Cecum-63: cecal contents sampled on day 63. Only pathways with significant differences between groups (*p* < 0.05) are shown, * *p* < 0.05 compared with the Ctrl group.

In mid-term (31 d) fecal samples and 63 d ileal contents, no significant differences were observed in any functional pathways between the Test and Ctrl groups (*p* > 0.05). In 63 d fecal samples, three pathways—Biosynthesis of siderophore group nonribosomal peptides, Cyanoamino acid metabolism, and Flagellar assembly—were significantly up-regulated in the Test group (*p* < 0.05) (Figure 9). In caecal contents at 63 d, three pathways—Biosynthesis of type II polyketide products, Polyketide sugar unit biosynthesis, and Steroid biosynthesis—were significantly up-regulated in the Test group (*p* < 0.05), while a total of 84 pathways were significantly down-regulated (Figure 9). These down-regulated pathways included those involved in carbohydrate metabolism, such as the Glycolysis/Gluconeogenesis, Pyruvate metabolism, and Pentose phosphate pathways; amino acid metabolism, including Alanine, aspartate, and glutamate metabolism, Arginine and proline metabolism, and Glycine, serine, and threonine metabolism; lipid metabolism, such as Fatty acid biosynthesis, Glycerophospholipid metabolism, and Linoleic acid metabolism; nucleotide metabolism, including Purine metabolism and Pyrimidine metabolism; energy metabolism, represented by Nitrogen metabolism; cofactor and vitamin metabolism, such as Nicotinate and nicotinamide metabolism, Pantothenate and CoA biosynthesis, and Riboflavin metabolism; genetic information processing, including DNA replication, Base excision repair, and Ribosome; environmental information processing, such as the Phosphotransferase system (PTS), Bacterial chemotaxis, and the Two-component system; xenobiotic degradation and metabolism, including Benzoate degradation, Bisphenol degradation, and Chloroalkane and chloroalkene degradation; and cellular processes and signal transduction, such as the NOD-like receptor signaling pathway and Insulin signaling pathway.

## 4. Discussion

The promoting effects of probiotics on growth performance of broilers have been documented in numerous studies. Nevertheless, no significant improvement in growth performance was observed in the present study, which could be attributed to multiple factors, including the initiation time and administration mode of probiotic supplementation, strain combination ratio, dosage, host breed, as well as shifting patterns of gut microbial communities.

Growth-promoting effects of probiotics vary markedly across different growth phases of broilers. A meta-analysis evaluating dietary *Lactobacillus* supplementation in broilers demonstrated that *Lactobacillus* exerted stronger growth-promoting effects during the early-growth phase than the late-growth phase. Specifically, the standardized mean difference (SMD) for average daily gain (ADG) in the early phase reached 1.05 (*p* < 0.001), which was substantially higher than that in the late phase (SMD = 0.52, *p* = 0.090) [24]. Such phase-dependent differences can be mechanistically explained as follows: the dominant gut microbiota of young broilers is still under establishment and vulnerable to external disturbances. Disruption of gut homeostasis in early life impairs the growth and health of chickens. Accordingly, probiotic supplementation at this developmental window facilitates the maintenance of gut microbial balance and thus yields more pronounced benefits. In this study, however, probiotics were first administered at day 22, when the intestinal microbiota of broilers had already formed a relatively stable configuration. Limited niche space and colonization efficiency for exogenous probiotics consequently weakened their growth-promoting potency.

Administration mode is another critical determinant of probiotic efficacy in broilers. Avberšek et al. [22] reported that identical probiotic products exerted divergent influences on production performance and cecal microbiota when delivered via drinking water versus feed, and such effects exhibited considerable variability across farms and experimental batches. By comparing three early-supplementation regimens (single-dose, intermittent, and continuous), Guo et al. [25] demonstrated that continuous early supplementation of *Limosilactobacillus fermentum* most effectively improved broiler production performance via ameliorating intestinal morphology, barrier function, and microbiota. In our trial, an intermittent drinking-water regimen was adopted: probiotics were supplied for four consecutive days followed by a three-day withdrawal period. Although this regimen facilitates practical farm management, it may cause periodic fluctuations of viable probiotic concentrations in the gut and fail to sustain stable probiotic benefits.

Multiple studies have illustrated the prominent synergistic advantages of multi-strain compound probiotics, which outperform single-strain probiotics in improving host growth performance, modulating immunity, and reshaping gut microbiota [26,27,28]. Nevertheless, the multi-strain probiotic preparation used in this study (*Lactobacillus salivarius*:*L. acidophilus*:*L. reuteri* = 38:9:1, final concentration 1.45 × 10^9^ CFU/L) did not produce significant growth-enhancing effects. Kim et al. [29] conducted side-by-side comparisons between single-and mixed-strain probiotics in both laying hens and broilers, and found divergent probiotic responses between the two poultry types. In broiler chicks, body weight gain in the multi-strain probiotic group was even significantly lower than that in the control and antibiotic groups. Furthermore, the ratio and dosage of compound probiotics also shape their biological outcomes in broilers. Xie et al. [30] investigated how different strain ratios affected growth performance, slaughter performance traits, and immune indices in broilers under identical inclusion dosage, and observed obvious discrepancies among formulations; the optimal performance was achieved at the ratio of *Bacillus subtilis*:*Saccharomyces cerevisiae*:*Lactobacillus acidophilus*:*Bifidobacterium animalis* = 1:2:1:1. Li et al. [31] supplemented compound probiotics at four graded doses (0, 500, 1000, 2000 g/t). The 2000 g/t group exhibited highly significant elevation in average daily gain and highly significant reduction in feed-to-gain ratio, together with the highest immune-organ indices and serum IgA and IgM concentrations among all treatments. Collectively, although each constituent strain in our compound preparation has proven probiotic efficacy when applied individually, interactions among mixed strains are complex. Beneficial effects of multi-strain preparations are not simple additive outcomes of single-strain functions. Antagonistic interactions may exist among certain strains; alternatively, the selected strain ratio, dosage, and host breed (Lingnan yellow-feathered broilers) in this study might restrict the full realization of growth-promoting potential.

In this study, supplementation of compound probiotics did not significantly alter alpha diversity in ileal and fecal microbiota. Although cecal microbial richness was significantly elevated, community evenness remained unchanged, indicating that this probiotic mixture failed to enhance overall gut microbial diversity in Lingnan yellow-feathered broilers. At the genus level, relative abundance of the beneficial genus *Lactobacillus* was significantly down-regulated in the ileum of probiotic-treated birds, which may partly explain why production performance in the Test group was not superior to that of the Ctrl group. At the functional level of KEGG pathways, ileal microbial functions showed no substantial shifts upon probiotic intervention. By contrast, as many as 84 KEGG pathways were significantly down-regulated in cecal microbiota, covering core functional categories including carbohydrate metabolism, amino acid metabolism, lipid metabolism, nucleotide metabolism, and genetic information processing. Down-regulation of carbohydrate-metabolism pathways (e.g., glycolysis/gluconeogenesis, pentose-phosphate pathway, pyruvate metabolism) suggests suppressed energy-metabolic activity of cecal microbes. Repression of genetic information processing pathways (e.g., DNA replication, ribosome, base-excision repair) implies diminished microbial proliferative capacity. Therefore, despite increased species richness, global metabolic functions of the cecal microbial community were broadly inhibited. It is well-established that cecal microbiota of broilers ferment non-starch polysaccharides (NSP) to generate short-chain fatty acids (SCFAs), which lower hind-gut pH, suppress pathogenic microbes [32,33], supply energy for intestinal epithelial cells, promote enterocyte proliferation, and enhance nutrient-absorptive capacity [34]. Widespread depression of microbial metabolic functions in the cecum would inevitably reduce nutritional and energetic contributions derived from microbial fermentation, which constitutes another plausible explanation for the lack of growth performance improvement.

Accumulating evidence indicates that the health-protective effects and growth-promoting effects of probiotics can be decoupled. Under heat-stress conditions, drinking-water supplementation of a dual-strain probiotic markedly reduced broiler mortality without significant changes in body weight gain or feed conversion ratio [35]. Similarly, *Lactobacillus plantarum* supplementation decreased mortality in pathogenic *Escherichia coli*-challenged broilers but failed to improve growth performance [36]. A broiler trial performed under high-altitude environments also demonstrated that probiotic supplementation mitigated mortality caused by ascites and coccidiosis, whereas weight gain and feed efficiency remained unaltered [37]. Consistent with these previous reports, although our compound probiotic preparation did not enhance production performance, it numerically increased villus-to-crypt ratios in the jejunum and ileum and significantly reduced the elimination rate in both the 43–63 d period and the entire experimental period (*p* < 0.05). Notably, eliminated birds in both groups consistently presented with persistent hypophagia, progressive emaciation, and lethargy—all of which are typical clinical manifestations of subclinical intestinal dysfunction and impaired nutrient absorption [38,39]. This observation suggests that probiotics do not necessarily elicit growth benefits; their actions can be primarily manifested in disease prevention and host intestinal health maintenance. The pronounced enrichment of *Akkermansia* in the cecum may partially account for the lowered elimination rate in the Test group. *Akkermansia muciniphila*, a novel mucin-degrading anaerobic bacterium, has been validated to confer benefits for intestinal-integrity maintenance, inflammation alleviation, and metabolic regulation [40,41]. After colonizing chicken gut, *A. muciniphila* activates the Wnt/β-catenin signaling pathway to accelerate intestinal epithelial cell proliferation, repairs anatomical and morphological intestinal damage induced by *Salmonella pullorum*, relieves inflammation, and restores body weight in infected chicks. Meanwhile, this bacterium elevates goblet-cell numbers, up-regulates Mucin2 and Tff2 expression, improves colon length and crypt depth, and increases proliferating-cell nuclear-antigen levels [42]. Another study revealed that oral gavage of *A. muciniphila* stabilizes gut microbiome composition in broilers, up-regulates innate-immune and antiviral defense responses, and alleviates lipopolysaccharide (LPS)-triggered immunosuppression [43]. Additionally, significant down-regulation of the NOD-like receptor signaling pathway (an inflammation- and immunity-related pathway) in the cecum provides another potential mechanism for the reduced elimination rate in the Test group. Excessive activation of the NOD-like receptor signaling pathway is tightly linked to aggravated inflammatory responses, intestinal tissue injury, and elevated mortality in broilers. In *Clostridium perfringens*-induced necrotic-enteritis models, hyper-activation of the NLRP3 inflammasome (a member of NOD-like receptor family) drives massive pro-inflammatory-cytokine release, resulting in mucosal destruction and increased mortality. Suppression of this pathway mitigates intestinal pathological lesions and reduces necrotic-enteritis-associated mortality [44]. Comparable patterns have been documented under light-stress, heavy-metal intoxication, and Salmonella infection challenges in broilers: aberrant over-activation of NOD-like receptor signaling amplifies inflammatory cascades, whereas moderate down-regulation alleviates tissue injury and mortality risk [45,46,47]. Collectively, over-activation of NOD-like receptor signaling correlates closely with inflammation, tissue damage, and higher mortality in broilers; conversely, down-regulation of this pathway helps relieve inflammatory injury and lower mortality risk. Taken together, the up-regulated cecal Akkermansia abundance and repressed NOD-like receptor signaling pathway in the Test group imply that probiotic supplementation may strengthen intestinal-barrier function and mitigate gut inflammation, thereby decreasing culling risk caused by inflammation-related diseases during later-phase rearing of broilers. It should, however, be acknowledged that this inference is derived from metagenomic functional prediction rather than direct measurement of immunological markers; while this finding provides a plausible mechanistic link between probiotic-induced microbiota shifts and reduced eliminations, direct measurements of intestinal and systemic inflammatory markers (e.g., IL-1β, IL-6, TNF-α, IgA, and tight-junction gene expression) are warranted in future studies to validate this interpretation.

## 5. Conclusions

Overall, the compound probiotic failed to improve the growth performance of Lingnan yellow-feathered broilers, which may be attributed to the mismatch between the probiotic’s initiation timing, intermittent drinking water administration, strain ratio, dosage, and this broiler strain. Meanwhile, the decreased ileal *Lactobacillus* abundance and the significant down-regulation of 84 core functional pathways covering carbohydrate, amino acid, lipid, nucleotide metabolism and genetic information processing in cecal microbiota further limit its growth-promoting effect. However, it significantly reduced the elimination rate in broilers, which may be associated with enrichment of cecal beneficial *Akkermansia* and down-regulation of inflammatory-immune pathways such as the NOD-like receptor signaling pathway to enhance intestinal health resilience, suggesting its potential as a health regulator in antibiotic-free poultry production.

## Figures and Tables

**Table 1:** Ingredient, composition, and nutrient content of experimental diets (g/100 g).

Items	1~21 d	22~42 d	43~63 d
Ingredients			
Corn	58.70	65.00	68.50
Soybean meal	34.30	27.00	23.00
Corn protein meal	2.00	2.50	3.00
Soybean oil	1.00	1.50	1.50
Premix	4.00 ^1,2^	4.00 ^1,3^	4.00 ^1,4^
Total	100.00	100.00	100.00
Nutrient components			
Crude protein	21.03	18.56	17.31
Metabolizable energy (MJ/kg)	11.85	12.18	12.31
Calcium	1.00	0.90	0.85
Phosphorus (available)	0.35	0.30	0.27
Lysine	1.10 ^5^	0.95 ^5^	0.80 ^5^
Methionine	0.50 ^5^	0.47 ^5^	0.42 ^5^
Methionine + Cystine	0.85 ^5^	0.80 ^5^	0.72 ^5^

^1^ For 1–21 d, 22–42 d, and 43–63 d broilers, the corresponding stage premixes adopted in the experiment were Qinbao 4101, Qinbao 4102, and Qinbao 4103 compound premixes, which are manufactured by Guangdong Xinnandu Feed Technology Co., Ltd. (Guangdong, China), respectively. ^2^ Per kilogram of diet for 1–21 d broilers, the premix provided the following nutrients and additives: VA 3600 IU; VD3 1400 IU; VE 15 mg; VB1 1.38 mg; VB2 4.0 mg; VB6 2.8 mg; calcium pantothenate 7.2 mg; nicotinamide 23.6 mg; folate 0.78 mg; Fe 26.4 mg; Cu 2.6 mg; Zn 24.2 mg; Mn 28.6 mg. ^3^ Per kilogram of diet for 22–42 d broilers, the premix provided the following nutrients and additives: VA 10000 IU; VD3 5000 IU; VE 8 mg; VB2 2.0 mg; VB6 2.0 mg; VB12 25 μg; Fe 75 mg; Cu 25 mg; Zn 120 mg; Mn 120 mg. ^4^ Per kilogram of diet for 43–63 d broilers, the premix provided the following nutrients and additives: VA 10000 IU; VD3 5000 IU; VE 6 mg; VB2 2.0 mg; VB6 1.0 mg; Fe 75 mg; Cu 25 mg; Zn 120 mg; Mn 150 mg. ^5^ Lysine, methionine, and methionine + cystine were provided as synthetic amino acids (Henan Tianqi Food Ingredients Co., Ltd., Henan, China) via the premix. Their listed levels are formulated values based on the analyzed amino acid contents of major ingredients, with synthetic amino acids supplemented to achieve the target values shown; they were not chemically measured in the final diets.

**Table 2:** Intestinal morphology of Lingnan yellow feather broilers.

Intestine	Index	Ctrl (n = 10)	Test (n = 10)
Jejunum	Villus height (mm)	1.422 ± 0.1189	1.369 ± 0.2485
	Crypt depth (mm)	0.3200 ± 0.05593	0.2781 ± 0.04268
	Villus-to-crypt ratio	4.529 ± 0.6564	4.946 ± 0.6710
Ileum	Villus height (mm)	1.059 ± 0.1557	1.101 ± 0.1933
	Crypt depth (mm)	0.2411 ± 0.02071	0.2419 ± 0.03325
	Villus-to-crypt ratio	4.420 ± 0.7511	4.647 ± 1.145

Values are presented as means ± standard deviation (SD, n = 10).

## Data Availability

Raw data are held by the author and may be available upon request.

## Supplementary Materials

The following supporting information can be downloaded at: https://www.mdpi.com/article/10.3390/ani16213385/s1, Table S1: Water consumption and estimated probiotic intake in the Test group during each experimental period; Table S2: Growth performance data during each experimental period.

## Abbreviations

The following abbreviations are used in this manuscript:

ADG	average daily gain
TWG	total weight gain
ADFI	average daily feed intake
TFC	total feed consumption
SR	survival rate
ER	elimination rate
FCR	feed conversion rate
SUN	serum urea nitrogen
CREA	serum creatinine
TG	serum triglyceride
CHO	serum total cholesterol
