# Supplementary material for: Modulation of Cecal Microbiota and Inflammatory-Immune Pathways Underlies the Reduced Eliminations but Unchanged Growth in Broilers Fed a Compound Probiotic
Source: Animals (Basel). 2026 Oct 27;16(21):3385. doi: 10.3390/ani16213385 (PMC13762181; doi:10.3390/ani16213385)
Supplement: Supplementary file 1 [file animals-16-03385-s001.zip › animals-4580078-supplementary.pdf]

**Supplementary Table S1.** Water consumption and estimated probiotic intake in the Test group during each experimental period

| **Period** | **Water consumption with probiotics (L)** | ***L. salivarius* added (×10⁹ CFU)** | ***L. acidophilus* added (×10⁹ CFU)** | ***L. reuteri* added (×10⁹ CFU)** | **Total probiotic intake (×10⁹ CFU)** | **Final probiotic concentration (×10⁹ CFU/L)** | **No. of birds at start of phase** | **Estimated probiotic intake per bird (×10⁹ CFU)** | **Estimated daily probiotic intake per bird (×10⁹ CFU)** |
|---|---|---|---|---|---|---|---|---|---|
| 1–21 d | 0 | 0 | 0 | 0 | 0 | 0 | 350 | 0 | 0 |
| 22–42 d | 852.86 | 984.07 | 227.09 | 26.20 | 1,237.36 | 1.45 | 349 | 3.55 | 0.29 |
| 43–63 d | 1,198.71 | 1,383.13 | 319.18 | 36.83 | 1,739.14 | 1.45 | 344 | 5.06 | 0.42 |
| entire period (1–63 d) | 2,051.57 | 2,367.20 | 546.28 | 63.03 | 2,976.51 | 1.45 | 350 | 8.60 | 0.36 |

**Supplementary Table S2.** Growth performance data during each experimental period

| | | Ctrl group（n=5） | Test Group（n=5） | P value |
|---|---|---|---|---|
| Average daily gain (ADG, g/d) | 1-21d | 16.26±0.1817 | 16.24±0.1817 | 0.8661 |
| | 22-42d | 27.34±1.445 | 25.78±0.5675 | 0.0548 |
| | 43-63d | 38.06±1.504 | 38.04±2.505 | 0.9882 |
| | entire period | 27.22±0.6686 | 26.70±0.8803 | 0.3226 |
| Total weight gain (TWG, kg) | 1-21d | 23.90±0.295 | 23.81±0.212 | 0.587 |
| | 22-42d | 39.46±2.254 | 36.96±0.9782 | 0.0525 |
| | 43-63d | 53.94±2.081 | 53.78±4.474 | 0.9426 |
| | entire period | 117.3±3.665 | 114.6±5.348 | 0.3723 |
| Average daily feed intake (ADFI, g/(bird·d)) | 1-21d | 26.22±0.4970 | 26.08±0.3421 | 0.6179 |
| | 22-42d | 66.68±2.594 | 63.82±2.141 | 0.0937 |
| | 43-63d | 100.6±2.297 | 101.2±5.726 | 0.8275 |
| | entire period | 64.48±1.291 | 63.70±2.338 | 0.5320 |
| Total feed consumption (TFC, kg) | 1-21d | 38.53±0.7050 | 38.20±0.6250 | 0.4587 |
| | 22-42d | 96.28±4.080 | 91.58±3.825 | 0.0972 |
| | 43-63d | 142.5±4.580 | 142.9±10.28 | 0.9377 |
| | entire period | 277.3±8.373 | 272.7±13.90 | 0.5424 |
| Feed conversion rate (FCR) | 1-21d | 1.61±0.01581 | 1.604±0.01817 | 0.5927 |
| | 22-42d | 2.442±0.04438 | 2.476±0.05857 | 0.3311 |
| | 43-63d | 2.644±0.05505 | 2.660±0.04848 | 0.6388 |
| | entire period | 2.366±0.02602 | 2.380±0.02915 | 0.4239 |
| Survival rate (SR, %) | 1-21d | 100±0.00 | 99.72±0.6261 | 0.3466 |
| | 22-42d | 99.44±0.7668 | 99.44±0.7668 | 0.9999 |
| | 43-63d | 99.40±1.342 | 99.72±0.6261 | 0.6418 |
| | entire period | 98.86±1.207 | 98.88±0.6261 | 0.9746 |
| Elimination rate(ER,%) | 1-21d | 0 | 0 | / |
| | 22-42d | 0.5714±0.7825 | 0.8613±0.7863 | 0.5751 |
| | **43-63d** | **6.793±0.8786** | **4.190±1.347** | **0.0068** |
| | entire period | **7.143±1.010** | **4.857±1.629** | **0.0285** |
